# Different molecular pathways are disrupted in Pyoderma gangrenosum patients and are associated with the severity of the disease

**DOI:** 10.1038/s41598-023-31914-z

**Published:** 2023-03-25

**Authors:** Ronald Rodrigues Moura, Lucas Brandão, Chiara Moltrasio, Almerinda Agrelli, Paola Maura Tricarico, Carlo Alberto Maronese, Sergio Crovella, Angelo Valerio Marzano

**Affiliations:** 1 Department of Advanced Diagnostics, Institute for Maternal and Child Health-IRCCS “Burlo Garofolo”, 34137 Trieste, Italy; 2 Department of Pathology, Federal University of Pernambuco, Recife, 50670-901 Brazil; 3 Dermatology Unit, Fondazione IRCCS Ca’ Granda Ospedale Maggiore Policlinico, Milan, Italy; 4 Laboratory of Nanostructured Materials (LMNANO), Center for Strategic Technologies Northeastern (CETENE), Av. Prof. Luís Freire, 1-Cidade Universitária, Recife, 50740-545 Brazil; 5 Department of Physiopathology and Transplantation, Università Degli Studi Di Milano, Via Pace 9, 20122 Milan, Italy; 6 Biological Science Program, Department of Biological and Environmental Sciences, College of Arts and Sciences, Qatar University, Doha, State of Qatar

**Keywords:** Genetics, Immunology, Pathogenesis

## Abstract

Pyoderma gangrenosum (PG) is a rare inflammatory skin disease classified within the spectrum of neutrophilic dermatoses. The pathophysiology of PG is yet incompletely understood but a prominent role of genetics facilitating immune dysregulation has been proposed. This study investigated the potential contribution of disrupted molecular pathways in determining the susceptibility and clinical severity of PG. Variant Enrichment Analysis, a bioinformatic pipeline applicable for Whole Exome Sequencing data was performed in unrelated PG patients. Eleven patients were enrolled, including 5 with unilesional and 6 with multilesional PG. Fourteen pathways were exclusively enriched in the “multilesional” group, mainly related to immune system (i.e., type I interferon signaling pathway), cell metabolism and structural functions. In the “unilesional” group, nine pathways were found to be exclusively enriched, mostly related to cell signaling and cell metabolism. Genetically altered pathways involved in immune system biology and wound repair appear to be nodal pathogenic drivers in PG pathogenesis.

## Introduction

Pyoderma gangrenosum (PG) is a rare inflammatory skin disease classified within the spectrum of neutrophilic dermatoses (ND). Clinically, PG is characterized by painful ulcers with undermined, erythemato-violaceous edges^1,2^. In addition to the classic ulcerative form which accounts for approximately 85% of cases^1^, several other variants have been recognized, including: (i) bullous, (ii) pustular, (iii) vegetative (iv) peristomal, (v) genital, (vi) infantile and (vii) extracutaneous^2^, with individual cases sometimes transitioning from one variant to the other^1^.

PG can either occur as an isolated dermatosis or in association with systemic conditions such as inflammatory bowel diseases (IBD), arthritis and haematological disorders, including paraproteinemia and malignancies. In a third, more restricted subset of cases, PG represents a component of distinct autoinflammatory syndromes^3^ namely PAPA (Pyogenic Arthritis, PG and acne), PASH (PG, acne and suppurativa hidradenitis), PASS (PG, acne conglobata, suppurativa hidradenitis, seropositive spondyloarthropathies) PAPASH (pyogenic arthritis, pyoderma gangrenosum, acne and hidradenitis suppurativa), PsAPASH (Psoriatic arthritis, PG, acne, suppurativa hidradenitis) and SAPHO (synovitis, acne, pustulosis, hyperostosis, osteitis)^4–7^.

Different sets of criteria have been proposed to assist the diagnosis of PG^8–10^, the most recent is the PARACELSUS score^10^, considered as highly effective and sensitive; however, there is an ongoing debate on which the best clinical algorithm to define PG severity.

The pathophysiology of PG is yet incompletely understood. Available evidence points towards the contribution of a genetically predisposed background facilitating the dysregulation of innate and adaptive immune responses^11^, with the pilosebaceous unit as the putative initial target^12,13^.

The role of genetics in PG pathogenesis is perhaps best illustrated in PG-associated genetic syndromes. Indeed, causative variants in *PSTPIP1*, *MEFV*, *NLRP3*, *NLRP12* and *NOD2* genes, have been hypothesized to promote an exaggerated IL-1β release, thus initiating and amplifying a wide variety of effects linked to innate immunity, tissue damage and autoinflammation^14^. Interestingly, despite affecting genes related to other autoinflammatory diseases, causative genetic defects are mostly patients’ specific private variants.

To the best of our knowledge, genetic findings have yet to shed light on the biological pathways involved in the disease.

Through Variant Enrichment Analysis (VEA), a bioinformatic pipeline applicable for Whole Exome Sequencing (WES) data, this study aimed at disclosing the potential role of altered molecular pathways in determining the clinical severity of unrelated PG cases.

## Results

### Patients

Eleven PG patients (6 men, 5 women) were enrolled. Clinical features of PG patients are summarized in Supplementary Table S1. One had PG in the setting of SAPHO while another had PASS syndrome.

Mean age at PG diagnosis was 45.6 years. Ten patients presented with classic ulcerative PG while one had bullous features. Lower limb involvement was documented in 8 patients, with the remaining three showing PG lesions on the trunk (1), the upper limbs (1) and the genitals (1). Five patients had unilesional PG. In these cases there was only one ulcer, which was hallmarked by reddish erythema, oedema (3 out of 5 patients) and typical border undermining. The other six patients suffered from multilesional PG (more than two ulcers), with multiple ulcers characterized by pronounced erythema, oedema, and ulcer border elevation.

Almost all patients were treated with topical therapy, mainly represented by high potency corticosteroids; intralesional treatment was administered in association to topical therapy in one case. Systemic immunosuppressants were also given to all patients, eight of whom also received systemic immunomodulators and biologic agents, including anti-tumour necrosis factor (TNF)-α (6) and anti-IL-17A (2) agents.

Five patients also underwent wound care treatments including compression, use of impregnated gauze and epidermal grafting.

Complete ulcer healing was reached in 8 patients. Two achieved partial remission while one died due to Coronavirus Disease 2019 (COVID-19).

### Exome analyses

**Figure 1:**
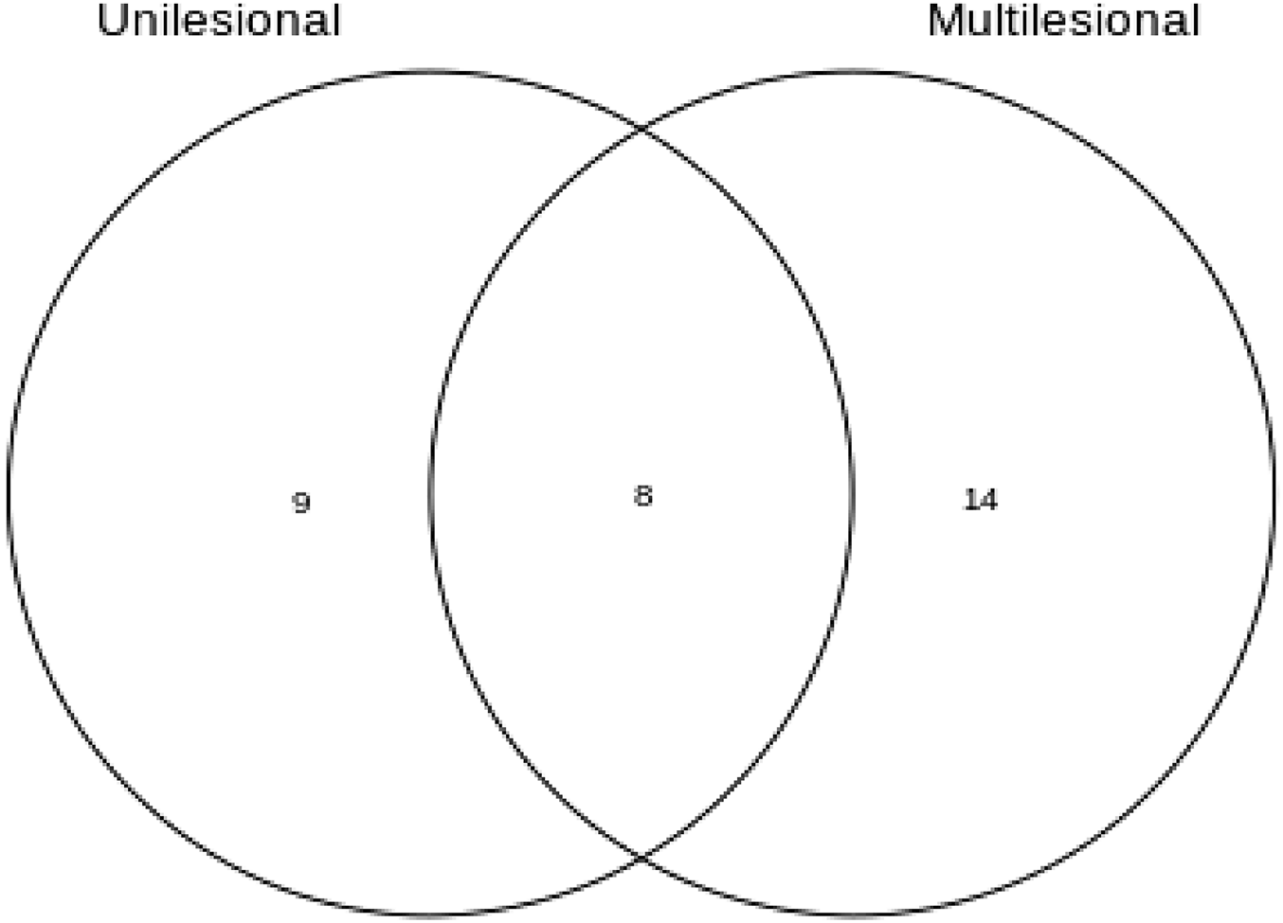
Veen diagram analysis of Variant Enrichment Analysis (VEA) of 11 exomes from patients with Pyoderma gangrenosum classified by disorder’s severity. 8 enriched pathways (EP) are shared between “unilesional” and multilesional. 9 and 14 EP exclusive in “unilesional” and multilesional respective.

Whole exomes of 11 patients diagnosed with PG and its associated syndromes (one patient with SAPHO and one with PASS syndrome), stratified according to disease severity, were analysed by Variant Enrichment Analysis (VEA). Detailed descriptive information about individual exomes and stratification according to disease severity is reported in our complete and interactive website report [https://davinci.biohub.solutions/pyoderma/]. Here, we describe our WES analysis approach focusing on VEA findings for both “unilesional” and “multilesional” groups. Shared and exclusive enriched pathways (eEP) of the two groups were identified. Notably, only variants shared by all individuals of each group were included in the VEA. A Venn Diagram Analysis showing the number of exclusive and shared pathways between the groups is displayed on Fig. 1, as well Table 1 contains detailed information about the exclusive pathways.

**Table 1:** List of exclusive enriched variant pathways in patients diagnosed with Pyoderma gangrenosum according to severity of disease.

Disease severity	Reactome ID	Pathway name	OR	p. Adjust	ImportantVar
Multilesional	R-HSA-114508	Effects of PIP2 hydrolysis	1.64	0.0095	2
	R-HSA-1236977	Endosomal/Vacuolar pathway	1.71	0.0000	40
	R-HSA-1489509	DAG and IP3 signaling	1.68	0.0001	3
	R-HSA-164940	Nef mediated downregulation of MHC class I complex cell surface expression	4.54	0.0000	9
	R-HSA-2172127	DAP12 interactions	3.32	0.0000	39
	R-HSA-2871809	FCERI mediated Ca + 2 mobilization	1.61	0.0201	3
	R-HSA-5083629	Defective POMT2 causes MDDGA2. MDDGB2 and MDDGC2	3.47	0.0104	2
	R-HSA-5083633	Defective POMT1 causes MDDGA1. MDDGB1 and MDDGC1	3.47	0.0104	2
	R-HSA-5218921	VEGFR2 mediated cell proliferation	1.88	0.0032	2
	R-HSA-5607763	CLEC7A (Dectin-1) induces NFAT activation	2.08	0.0011	1
	R-HSA-5661270	Formation of xylulose-5-phosphate	3.51	0.0143	0
	R-HSA-70895	Branched-chain amino acid catabolism	2.05	0.0371	4
	R-HSA-9026762	Biosynthesis of maresin conjugates in tissue regeneration (MCTR)	6.84	0.0442	0
	R-HSA-909733	Interferon alpha/beta signaling	1.53	0.0000	57
Unilesional	R-HSA-163615	PKA activation	1.96	0.0146	1
	R-HSA-171319	Telomere Extension By Telomerase	3.72	0.0000	2
	R-HSA-187037	Signaling by NTRK1 (TRKA)	1.54	0.0039	10
	R-HSA-427975	Proton/oligopeptide cotransporters	3.58	0.015	3
	R-HSA-5083625	Defective GALNT3 causes familial hyperphosphatemic tumoral calcinosis (HFTC)	1.63	0.0000	224
	R-HSA-5083632	Defective C1GALT1C1 causes Tn polyagglutination syndrome (TNPS)	1.63	0.0000	224
	R-HSA-5083636	Defective GALNT12 causes colorectal cancer 1 (CRCS1)	1.62	0.0000	224
	R-HSA-5621480	Dectin-2 family	1.64	0.0000	225
	R-HSA-977068	Termination of O-glycan biosynthesis	1.64	0.0000	224

### Exclusive enriched pathways from “multilesional” group

Fourteen pathways were exclusively enriched in the “multilesional” group as shown in Table 1. These pathways were categorized into three major groups: pathways related to **Immune System** (R-HSA-9026762, R-HSA-164940, R-HSA-2172127, R-HSA-5607763, R-HSA-1236977, R-HSA-909733); **Cell Metabolism** (R-HSA-5218921, R-HSA-5661270, R-HSA-1489509, R-HSA-114508, R-HSA-2871809); and **Structural Functions** (R-HSA-5083629, R-HSA-5083633, R-HSA-70895).

R-HSA-164940 (Nef mediated downregulation of MHC class I complex cell surface expression) carried the highest Odd Ratio (OR) values (4.54) within the pathways presenting ImportantVars. In addition, the R-HSA-909733 (Interferon alpha/beta signaling pathway), the R-HSA-1236977 (Endosomal/Vacuolar pathway), the R-HSA-2172127 (DAP12 interactions pathway) showed an elevated number of important variations (impact or functional protein alteration).

### Exclusive enriched pathways from “unilesional ” group

In the “unilesional ” group, nine pathways were found, and they can be divided into four categories**: Immune System** (R-HSA-5621480); **Cell Metabolism** (R-HSA-171319, R-HSA-427975, R-HSA-977068); **Cell Signaling** (R-HSA-163615, R-HSA-187037); and **Disease Related** (R-HSA-5083625, R-HSA-5083632, R-HSA-5083636).

The R-HSA-171319 (Telomere Extension by Telomerase) and R-HSA-427975 (Proton/oligopeptide cotransporters) showed the highest OR values, 3.72 and 3.58, respectively, whereas the pathways related to Termination of O-glycan biosynthesis: R-HSA-977068, R-HSA-5083625, R-HSA-5083632, R-HSA-5083636 carried several ImportantVars: 225, 10 and 224, respectively.

### Enriched pathways shared by the “multilesional” and “unilesional” groups

Eight enriched pathways were shared by both the “multilesional” and the “unilesional” group, as shown in Table 2. R-HSA-450302 and R-HSA-983695 belong to the major group “**Immune System**”. Of the other six each falls into a different category: **Metabolism** (R-HSA-189483), **Cell cycle** (R-HSA-2995410), **Hemostasis** (R-HSA-418457), **Disease** (R-HAS-5210891), **Metabolism of proteins** (R-HSA-8876725) and **Gene expression transcription** (R-HAS-8941284).

**Table 2:** List of enriched variant pathways shared by “multilesional” and “unilesional” patients diagnosed with Pyoderma gangrenosum.

Reactome ID	Pathway name
R-HSA-189483	Heme degradation
R-HSA-2995410	Nuclear Envelope (NE) Reassembly
R-HSA-418457	cGMP effects
R-HSA-450302	Activated TAK1 mediates p38 MAPK activation
R-HSA-5210891	Uptake and function of anthrax toxins
R-HSA-8876725	Protein methylation
R-HSA-8941284	RUNX2 regulates chondrocyte maturation
R-HSA-983695	Antigen activates B Cell Receptor (BCR) leading to generation of second messengers

## Discussion

Through a novel bioinformatic approach this study provided insights on the main biological pathways disrupted by genetic defects in a small cohort of unrelated PG patients.

Specifically, we focused on the different pathways involved in “multilesional” and “unilesional” PG, using these groups as a proxy for disease severity.Both in the “multilesional” and “unilesional” groups, VEA workflow applied to WES data showed immune and wound repair processes in PG susceptibility and severity modulation.

Interestingly, most of the pathways, including those related to extracellular matrix (ECM) remodeling, type I interferons and DAP12 (DNAX activating protein of 12 kDa) Interaction pathways herein discussed have been documented to encompass genes differentially expressed in cutaneous PG samples respective to healthy control skin, further crediting their contribution to the disease pathophysiology^15^.

Among the seven Immune System pathways disrupted in the “multilesional” group exclusively, Type I interferons (IFN) (R-HSA-909733) are of particular interest, representing key cytokines for the development of innate and adaptive immune responses, with important roles in host defense against viral infections and autoimmunity. Canonical type I IFN signaling activates the Janus kinase (JAK)–signal transducer and activator of transcription (STAT) pathway and is known to be involved in pathogenesis of PG. Our results fall in line with this premise and, indeed, JAK inhibition is shaping up as a compelling treatment option for PG, although its long-term safety is still under investigation^16^.

Further, the CLEC7A (Dectin-1) Induces NFAT Activation pathway (R-HSA-5607763) may also fit in PG complex pathogenesis due to its association with the extracellular release of interleukin (IL)-2, a key mediator of regulatory T cells (Treg cells) functions^17–19^. Tregs cells play a central role in preventing autoimmune and autoinflammatory processes and in fact an imbalance between Tregs and T helper (Th)17 cells has been detected in PG^20^.

Acting upstream of T cell polarization, the major histocompatibility complex class I (MHC-I) antigen presentation plays a key role in alerting the immune system to cells infected; a downregulation of MHC-I molecules at the cell surface decreases the ability of CD8^+^ T cells to recognize peptides with a consequent impairment in immune responses^21,22^. The Nef Mediated Downregulation of MHC- I complex cell surface expression (R-HSA-164940) and the Endosomal/Vacuolar (R-HSA-1236977) pathways are necessary for antigen processing by MHC-I, suggesting that MHC-I pathway could play a role in PG pathogenesis. Furthermore, the endosomal/vacuolar pathway (R-HSA-1236977) is related to autophagy, a process that plays a housekeeping role in maintaining cellular homeostasis and protects against genome instability^23^. Dysregulated or absent autophagy results in a wide range of conditions, including inflammatory and immune-mediated skin diseases^24^. In addition, defective autophagy in endothelial cells determines excessive transendothelial migration (TEM), with subsequent neutrophil infiltration and tissue damage^25^—both key events in PG pathophysiology.

Two additional pathways may have effect on innate immune cell function, i.e., the Maresin Conjugates in Tissue Regeneration (MCTR) (R-HSA-9026762) biosynthetic pathway and the DAP12 Interaction pathway (R-HSA-2172127).

MCTR is a family of recently identified macrophage-derived mediators promoting the resolution of inflammatory processes, through a wide variety of biological actions. Although their role in the skin has been poorly elucidated, a variety of experimental models has demonstrated their effects in alleviating neutrophil infiltration, reducing IL-17A levels^26^, stimulating M2-macrophage polarization^27^, hastening bacterial clearance as well as the repair and regeneration of damaged tissue. Accordingly, the disruption of their biosynthetic pathway (R-HSA-9026762) may contribute to the non-healing nature of PG ulcers^28^.

DAP12 is involved in the transduction of a wide variety of activating signals in neutrophils, monocytes/macrophages, natural killer (NK) and dendritic cells (DCs)^29^. Interestingly, in a landmark study comparing gene expression between PG and healthy control skin, several differentially expressed genes were noted to be associated with the DAP12 Interactions pathway (R-has-2172127)^15^. Although the latter may be but the reflection of PG cutaneous *milieu*, our results together with the pathway’s pivotal role in myeloid cells—mediating the production of pro-inflammatory cytokines, chemokines, reactive oxygen species, degranulation of neutrophilic granules, and phagocytosis^30^—support the idea of its contribution to the exaggerated inflammatory response observed in PG.

As most of these pathways also influence the clearance of bacterial infections, it is tempting to speculate that their disruption may translate into greater susceptibility to superinfections, prolonging/complicating PG course and adding a further layer of complexity to their biological meaning.

Considering the five Cell Metabolism pathways, their functions were related with cell survival, proliferation, migration, and vascular permeability (R-HSA-5218921)^31^; cellular respiration and catabolism (R-HSA-5661270 and R-HSA-70895)^32^; and signal transduction (R-HSA-1489509 and R-HSA-114508)^33,34^, all of which may have major repercussions on wound healing and therefore PG pathophysiology.

Regarding the two pathways associated with Structural Functions, they were mostly related with defective assembly of the extracellular matrix (R-HSA-5083629 and R-HSA-5083633)^35^. In the ECM pathway, a key role is played by metalloproteinases (MMPs), zinc-dependent extracellular proteases synthesized by inflammatory cells such as neutrophils that break down the ECM to enable tissue remodeling and wound repair^36^. MMP-9 and to a lesser extent MMP-2 have been found in the stroma of PG lesions^37^ and it has been postulated that an ongoing overexpression of MMPs by neutrophils within PG lesions, could promote prolonged ulceration.

Concerning the pathways enriched in the “unilesional” group exclusively, the Dectin-2 family Pathway (R-HSA-5621480) was the only one related to immune system. C-type lectin-like receptors (CLRs) of the “Dectin family” represent a family of transmembrane pattern recognition receptors (PRRs), mainly expressed by myeloid cells where they induce the production and release of cytokines like TNFα and IL-6, via nuclear factor-κB (NF-κB). Furthermore, these receptors are also involved in cutaneous wound healing by regulating neutrophil extracellular traps (NET)osis. In the context of PG, an impairment of this pathway could both promote an increased inflammatory response and affect the wound healing process of lesions through regulation of neutrophilic responses^38^.

Among the three pathways related to Cell Metabolism, the Telomere Extension by Telomerase pathway (R-HSA-171319), which participates both in endothelial cell proliferation and wound healing, and the Termination of O-glycan biosynthesis pathway (R-HSA-977068) are of keen interest. O-glycosylation is a post-translational modification that occurs after the protein has been synthesised, adding several different sugars to the serine or threonine residues^39^. O-glycans play various functions including cells trafficking in the immune system, recognition of foreign material, control of cell metabolism and epithelial differentiation^40^. It has been shown that alterations in O-glycosylation are important in the development of several diseases, including immune disorders, and based on its important functions it could also be involved in the susceptibility and occurrence of PG.

Concerning pathways related to Cell Signalling, we found protein kinase A (PKA) activation (R-HSA-163615) and Signaling by NTRK1 (TRKA) (R-HSA-187037). This latter is involved in skin wound healing through activation of extracellular signal-regulated kinase (ERK) and tyrosine kinases (TRK) receptors. Indeed, in a recent study, Chakrabarti et al.^41^, demonstrated that faster wound healing results from an increased re-epithelialization and granulation tissue formation due to the presence of proteins such as TrkA, p-TrkA, ERK1/2, p-ERK1/2 and NF-kβ. These results allow us to assume that an alteration of this pathway could be responsible for slower wound repair in PG cases.

Our study presents two limitations: the first one is the small sample size; the second one is related to not having compared our findings with other omics data, in particular, transcriptomics and proteomics from the skin.

In conclusion, we described altered biological pathways in PG patients, particularly involved in immune system, in neutrophils-related inflammation and in wound repair, confirming that these processes are nodal pathogenic drivers in PG pathogenesis.

## Materials and methods

### Patients

Saliva samples were collected from unrelated PG patients attending the Dermatology Unit of IRCCS Cà Granda Ospedale Maggiore Policlinico, Milan, Italy from January 2015 to January 2022. The study was approved by the Area B Milan Ethics Committee (protocol No. 487_2020).

All patients provided written informed consent to be included in the study. All research has been performed in accordance with the Declaration of Helsinki. PG diagnosis followed the PARACELSUS criteria^10^. An accurate family history was collected to rule out PG and PG-associated systemic conditions in the patients’ relatives.

### Exome analysis

The genomic DNAs were extracted from saliva samples using Oragene-DNA kit (Oragene DNA OG 500, Ottawa, Canada) according to manufacturers’ instructions. Agarose gel and Qubit (Invitrogen, Oregon, USA) evaluated the quantity and quality of the DNA extraction. Whole exome sequencing (WES) was performed with the Illumina® Exome SureSelect V6-post, producing pair-ended reads with a length of 151 base pairs (bp) and an estimated coverage of 150x. All sequencing procedures were outsourced by Macrogen®_._

The fastq files were then processed firstly by fastqQC (for quality control checking) (https://www.bioinformatics.babraham.ac.uk/projects/fastqc/)^42^ and later by TrimGalore for adapter trimming, and removal of low quality reads (Phred quality score < Q20 and read length < 25 bp) (http://www.bioinformatics.babraham.ac.uk/projects/trim_galore/)^43^. The remaining reads were mapped with Human Reference Genome (GRCh.38) using BWA software^44^; duplicated reads were detected and removed after mapping, using picard tools (https://broadinstitute.github.io/picard/)^45^, while base recalibration was performed using GATK (https://software.broadinstitute.org/gatk/)^46^. Variant Calling was made by strelka2^47^, using the exome mode, while variant annotation was carried out by ANNOVAR^48^. We used built-in R scripts to manipulate ANNOVAR output and proceed with per sample descriptive analysis, as well as the variant enrichment analysis (VEA)^49^. In brief, VEA predicts unexpected high levels of mutations in specific pathways, applying a False Discovery Rate (FDR) statistical model with non-Finnish European (NEF) population as genomic reference input and as a ratio of expected mutation levels. Important variants (ImportantVar) were defined as genetic variants with a positive damage prediction score, according to multiple algorithm prediction (CADD, SIFT, PolyPhen2, FATHMM, etc.), and variants with functional impact, such as non-synonymous, stop codon, and start codon variations^49^. Pathways with adjusted *p*-value lower than 0.05 were considered significant.

## Supplementary Information

Supplementary Table S1.

## Data Availability

The datasets generated and analysed during the current study are available in the BioProject repository under PRJNA801118 accession number. Each individual sample could be accessed at BioSamples (SAMN32262377, SAMN32262378, SAMN32262379, SAMN32262380, SAMN32262381, SAMN32262382, SAMN32262383, SAMN32262384, SAMN32262385, SAMN32262386, SAMN32262387).
