# Supplementary material for: Different molecular pathways are disrupted in Pyoderma gangrenosum patients and are associated with the severity of the disease
Source: Sci Rep. 2023 Mar 25;13:4919. doi: 10.1038/s41598-023-31914-z (PMC10039684; doi:10.1038/s41598-023-31914-z)
Supplement: Supplementary file 1 — Supplementary Table S1. [file 41598_2023_31914_MOESM1_ESM.pdf]

Table S1: Clinical features of patients with pyoderma gangrenosum

Pat.	Sex	Age at diagnosis	Diagnosis	Comorbidities	Type of PG	Target Ulcer Location	Target Ulcer Side	Target Ulcer Edema	Target Ulcer Erythema	Target Ulcer Erythema description (if erythema)	Target Ulcer Ulcer border elevation	Number of other ulcers	Prior topical treatments	Prior intralesional treatment	Prior systemic treatment	Outcome
1	F	49	PG	None	classic (ulcerative)	Lower Extremity	Left	Yes	Yes	Reddish	Yes	None (target ulcer is only ulcer)	Corticosteroids	None	Immunosuppresants	Complete healing
2	M	42	PG	Hypertension, hyperparathyroidism secondary to vitamin d deficiency, carpal tunnel syndrome, ocular hypertone	classic (ulcerative)	Lower Extremity	Right	Yes	Yes	Pronounced	Yes	2+ (multilesional PG)	Corticosteroids	Corticosteroids; antibiotics	Immunosuppresants; antibiotics; biologic agent (infliximab)	Complete healing
3	M	44	PG	None	classic (ulcerative)	Lower Extremity	Left	Yes	Yes	Reddish	Yes	None (target ulcer is only ulcer)	Corticosteroids	None	Immunosuppresants	Complete healing

Table S1: Clinical features of patients with pyoderma gangrenosum

4	M	32	PG	Hypertension	classic (ulcerative)	Lower Extremity	Trunk	No	Yes	Pronounced	Yes	2+ (multilesional PG)	Corticosteroids	None	Immunosuppresants; immunomodulators; biologic agent (adalimumab)	Not completely healed
5	M	25	PG	None	classic (ulcerative)	Upper extremity	Left	Yes	Yes	Pronounced	Yes	2+ (multilesional PG)	Corticosteroids	None	Immunosuppresants; biologic agent (adalimumab)	Complete healing
6	F	47	PASS	ankylosing spondylitis;PG;acne vulgaris;HS: PASS	classic (ulcerative)	Lower extremity	Left	No	Yes	Reddish	Yes	None (target ulcer is only ulcer)	Corticosteroids	None	Immunsuppresants; immunomodulators; biologic agents (TNF-inhibitors; IL-17 inhibitor)	Complete healing

Table S1: Clinical features of patients with pyoderma gangrenosum

7	F	65	PG	Lymphoplasmacytic lymphoma; primary myelofibrosis; essential thrombocytopenia; acquired Von Willebrand Syndrome	bullous	Lower extremities	Left	Yes	Yes	Pronounced	Yes	2+ (multilesional PG)	None	None	Immunsuppresants; immunomodulators; biologic agent (adalimumab)	Complete healing
8	M	53	PG/SAPHO	Synovitis; acne; pustulosis; hyperostosis; osteitis; uveitis; thyroiditis; PG;	classic (ulcerative)	Lower extremities	Left	No	Yes	Pronounced	Yes	2+ (multilesional PG)	None	None	Immunsuppresants; biologic agent (adalimumab)	DEATH
9	F	65	PG	None	classic (ulcerative)	Genitals	Center	Yes	Yes	Reddish	Yes	None (target ulcer is only ulcer)	None	None	Immunosuppresants; biologic agent (adalimumab)	Not completely healed

Table S1: Clinical features of patients with pyoderma gangrenosum

10	M	54	PG	None	classic (ulcerative)	Trunk	Center	No	Yes	Pronounced	Yes	2+ (multilesional PG)	Cortisteroids	None	Immunosuppresants	Complete healing
11	F	26	PG	anti-fospolipid antibody syndrome; SLE; recurrent deep vein thrombosis; hypovitaminosis. (heterozygous MTHFR mutation)	classic (ulcerative)	Lower extremities	Left	No	Yes	Severe (reddish)	Yes	None (target ulcer is only ulcer)	Dapsone,	None	Immunosuppresants; immunomodulators; biologic agent (adalimumab; IL-17 inhibitor)	Complete healing
